# A preliminary assessment of a stool-based microRNA profile for early colorectal cancer screening

**DOI:** 10.1038/s41598-025-14485-z

**Published:** 2025-08-05

**Authors:** Daniela A. R. Santos, Mariana Eiras, Miguel Gonzalez-Santos, Marlene Santos, Carina Pereira, Lúcio Lara Santos, Mário Dinis-Ribeiro, Luís Lima

**Affiliations:** 1 Experimental Pathology and Therapeutics Group, Research Centre of IPO Porto (CI- IPOP)/RISE@CI-IPOP (Health Research Network), Portuguese Oncology Institute of Porto (IPO Porto), Porto Comprehensive Cancer Centre (Porto.CCC), Porto, 4200-072 Portugal; 2 Faculty of Medicine of the University of Porto (FMUP), Porto, 4200-319 Portugal; 3 Institute of Biomedical Sciences Abel Salazar (ICBAS), University of Porto, Porto, 4050-313 Portugal; 4 School of Health, Polytechnic Institute of Porto, Rua Dr. António Bernardino de Almeida, 400, Porto, 4200-072 Portugal; 5 REQUIMTE/LAQV, Escola Superior de Saúde, Instituto Politécnico do Porto, Rua Dr. António Bernardino de Almeida, Porto, 4200-072 Portugal; 6 Molecular Oncology & Viral Pathology, IPO-Porto Research Centre (CI-IPO), Portuguese Institute of Oncology, Porto, 4200-072 Portugal; 7 Precancerous Lesions and Early Cancer Management Group, Research Centre of IPO Porto (CI-IPOP)/Rise@CI‐IPOP (Health Research Group), Portuguese Institute of Oncology of Porto (IPO Porto)/Porto Comprehensive Cancer Centre (Porto.CCC), Porto, 4200-072 Portugal; 8 Department of Surgical Oncology, Portuguese Institute of Oncology (IPO-Porto), Porto, 4200-072 Portugal; 9 Department of Gastroenterology, Portuguese Institute of Oncology (IPO-Porto), Porto, 4200-072 Portugal

**Keywords:** Colorectal cancer, Diagnostic markers

## Abstract

Colorectal cancer screening methods are well established worldwide as a fundamental pilar in CRC management, namely through non-invasive faecal occult blood testing. However, the limited sensitivity of faecal occult blood test for detecting precancerous lesions highlights the need to search for alternative tools, such as microRNAs (miRs). The main aim of this study was to identify stool-based miR profiles for early colorectal cancer detection. A panel with miR-21-5p, miR-199a-5p, and age showed a moderate performance for colorectal cancer detection (sensitivity: 88%). Additionally, miR-451a, miR-21-5p, miR-199a-5p, age, and gender showed high performance for discriminating high-grade dysplasia lesions (sensitivity: 91%). Moreover, when we obtained a positive result in either panel, we achieved a sensitivity of 96% for high-grade dysplasia lesions identification. Finally, when a negative result was obtained in these panels after a positive faecal occult blood test result, we accurately identified individuals without lesions. These findings demonstrate the potential of miR panels as non-invasive biomarkers for colorectal cancer and high-grade dysplasia lesions detection and could constitute a secondary screening method following a positive faecal occult blood test.

**Supplementary Information:**

The online version contains supplementary material available at 10.1038/s41598-025-14485-z.

## Introduction

Colorectal cancer (CRC) is the third most common cancer and the second leading cause of cancer-related death worldwide^1^. Several European countries have implemented population-based CRC screening programs, mainly based on a two-step approach: a non-invasive faecal occult blood test (FOBT) followed by a confirmatory colonoscopy, the gold standard method for screening^2,3^. Although FOBT is the most widely used test, its sensitivity is suboptimal (approximately 70–80% for malignant lesions and 30–40% for precancerous lesions), restricting the overall effectiveness of screening^4–6^. Colonoscopy remains the gold standard method for CRC screening due to its high sensitivity in detecting premalignant and cancerous lesions^3,7^. Nonetheless, colonoscopy is an invasive procedure, which presents inherent limitations, such as low patient adherence and high medical and economic burden^8,9^. These limitations underscore the crucial need to develop new, effective, and non-invasive screening tools to enhance CRC detection and reduce disease burden.

In this context, numerous molecules have been recently reported as potential new biomarkers for CRC screening, with microRNAs (miRs) emerging as some of the most promising candidates^10,11^. MiRs are short non-coding RNAs (18–24 nucleotides) that negatively regulate post-transcriptional gene expression^12^. Several studies have reported altered miR expression levels throughout CRC progression^11^. Furthermore, miR’s expression levels can be easily detected in biofluids such as stool, making them potential non-invasive biomarkers for CRC screening^13,14^.

Building on our team’s literature review on miRs associated with CRC early detection^15^ this study evaluates eight miR candidates (miR-21-5p, miR-92a-3p, miR-135b-5p, miR-451a, and miR-421—previously evaluated in stool samples—; and miR-139-3p, miR-4516, and miR-199a-5p—evaluated for the first time in stool samples) as potential non-invasive biomarkers for the early diagnosis of CRC and advanced precancerous lesions—specifically, their potential for improving CRC screening as a secondary screening tool following a positive FOBT result.

## Results

### Study population

Colonoscopy and histologic data were available for all 96 individuals, who had a median age of 60 years (min–max: 40–89 years) and were 53% male. Detailed clinical characteristics are shown in Table 1.

**Table 1:** Clinical characteristics of the 96 individuals included in this study.

Characteristics	No lesion	Low grade dysplasia	High grade dysplasia	Cancer
n	24	24	24	24
Age (median, min–max)	58 (50–72)	56 (49–74)	64 (40–85)	65 (51–89)
Male gender	38%	63%	58%	54%

### MicroRNA expression levels in stool samples

**Fig. 1:**
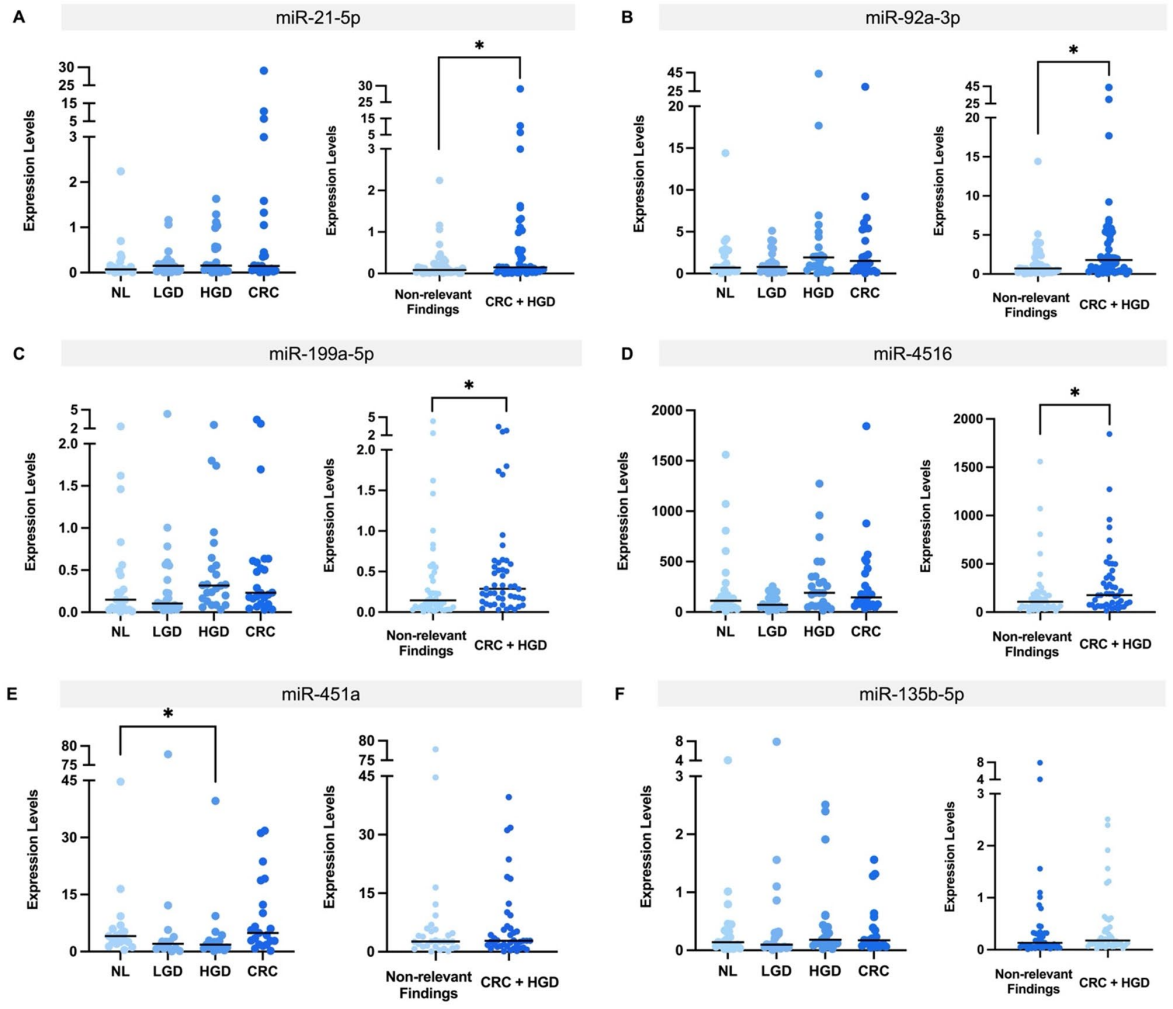
Stool expression levels of (**A**) miR-21-5p, (**B**) miR-92a-3p, (**C**) miR-199a-5p, (**D**) miR-4516, (**E**) miR-451a, and (**F**) miR-135b-5p, for different clinical groups and for non-relevant findings and HGD + CRC. *NL* No Lesion, *LGD* Low-Grade Dysplasia, *HGD* High-Grade Dysplasia, *CRC* Colorectal Cancer. *, *P* < 0.05.

To explore the clinical value of the analysed miRs in early CRC screening, their expression levels were assessed in stool samples using RT-qPCR (Fig. 1). Regarding the overall expression, miR-451a levels were significantly lower in HGD stool samples compared to NL (median: 1.818 vs. 4.088, *P* = 0.035). Furthermore, miR-21-5p (median: 0.151 vs. 0.086, *P* = 0.046), miR-92a-3p (median: 1.801 vs. 0.719, *P* = 0.044), miR-199a-5p (median: 0.287 vs. 0.145, *P* = 0.013), and miR-4516 (median: 176.3 vs. 107.1, *P* = 0.012), showed significantly higher expression levels in the CRC + HGD group compared to the non-relevant findings group.

### Clinical value of stool microRNAs in precancerous and cancerous lesions detection

ROC curve analysis was performed to assess the clinical value of stool miRs (Supplementary Table S1 and Fig. S1). All miRs showed poor discrimination between the LGD and NL groups. However, for HGD detection compared to NL, miR-451a demonstrated a moderate discrimination power (AUC: 0.706, 95% CI: 0.533–0.879), with good sensitivity (63%) and specificity (76%). Moreover, miR-21-5p, miR-92a-3p, miR-199a-5p, and miR-4516 exhibited acceptable discrimination for CRC and HGD identification (AUC: 0.620–0.650). Notably, miR-199a-5p showed the best performance for CRC + HGD detection compared to non-relevant findings (sensitivity: 71%; specificity: 60%).

### Stool-based microRNA combination for precancerous and cancerous lesions detection and their clinical applicability for screening

A stepwise multivariate logistic regression analysis with backward elimination was conducted to develop miR-based predictive models. Two different models combining miRs and clinical characteristics were generated (Panels A and B; Table 2).

**Table 2:** Discrimination capacity of stool-based MiR panels.

miR panels	Endpoint	AUC (95% CI)	Youden index	
			SENS (%) (95% CI)	SPEC (%) (95% CI)
Panel A	CRC + HGD^*a*^	0.729 (0.627–0.831)	69 (55–80)	68 (54–80)
	CRC^*b*^	0.799 (0.670–0.928)	88 (69–96)	48 (29–67)
	HGD^*b*^	0.707 (0.560–0.855)	75 (55–88)	48 (29–67)
Panel B	CRC + HGD^*a*^	0.650 (0.539–0.760)	88 (75–94)	40 (28–55)
	CRC^*b*^	0.662 (0.506–0.819)	79 (60–91)	48 (29–55)
	HGD^*b*^	0.831 (0.712–0.949)	91 (74–99)	70 (45–81)
Combination of panels A and B	CRC + HGD^*a*^	NA	92 (80–98)	40 (26–56)
	CRC^*b*^	NA	88 (68–97)	52 (31–73)
	HGD^*b*^	NA	96 (79–100)	52 (31–73)

*miR* microRNA, *HGD* High-Grade Dysplasia, *CRC* Colorectal Cancer, *AUC* Area under Curve, *NA* Not Applicable, *SENS* Sensitivity, *SPEC* Specificity, *CI* Confidence Interval. ^a^Negative Category: individuals with non-relevant findings in colonoscopy; ^b^ Negative Category: individuals with no lesions detected in colonoscopy. Panel A: miR-21-5p, miR-199a-5p, and age; Panel B: miR-21-5p, miR-199a-5p, miR-451a, age, and gender.

In detail, the combination of miR-21-5p, miR-199a-5p, and age (Panel A) demonstrated moderate discrimination for CRC identification compared to NL (sensitivity: 88%; AUC: 0.799, 95% CI: 0.670–0.928). The second model, combining miR-21-5p, miR-199a-5p, miR-451a, age, and gender (Panel B) was highly accurate in distinguishing HGD and NL (sensitivity: 91%; AUC, 0.831, 95% CI: 0.712–0.949) (Supplementary Fig. S2). Finally, when combining both panels (i.e., a positive result in at least one), diagnostic performance was maximised, showing a sensitivity of 92% for CRC + HGD detection compared to non-relevant findings, and 96% for discrimination between HGD and NL.

In order to assess the clinical applicability of miR-based predictive models following a positive FOBT result, positive and negative likelihood ratios were calculated (Table 3), and post-test probability was assessed.

**Table 3:** MiR panels likelihood ratios.

miRs combination	Endpoint	Positive likelihood ratio (95% CI)	Negative likelihood ratio (95% CI)
Panel A	CRC + HGD^*a*^	2.2 (1.2–4.0)	0.5 (0.3–0.8)
	CRC^*b*^	1.7 (1.0–2.9)	0.3 (0.1–1.1)
	HGD^*b*^	1.4 (0.8–2.3)	0.5 (0.2–1.6)
Panel B	CRC + HGD^*a*^	1.5 (1.0–2.1)	0.5 (0.1–0.9)
	CRC^*b*^	1.9 (0.8–2.8)	0.4 (0.1–1.4)
	HGD^*b*^	2.6 (1.3–5.2)	0.1 (0.0–0.6)
Combination of Panels A and B	CRC + HGD^*a*^	1.5 (1.1–2.2)	0.2 (0.0–0.8)
	CRC^*b*^	1.8 (1.0–3.6)	0.2 (0.0–1.0)
	HGD^*b*^	2.0 (1.1–3.7)	0.1 (0.0–0.7)

*miR* microRNA, *HGD* High-Grade Dysplasia, *CRC* Colorectal Cancer, *CI* Confidence Interval. ^*a*^ Negative Category: individuals with non-relevant findings in colonoscopy; ^*b*^ Negative Category: individuals with no lesions detected in colonoscopy. Panel A: miR-21-5p, miR-199a-5p, and age; Panel B: miR-21-5p, miR-199a-5p, miR-451a, age, and gender.

**Fig. 2:**
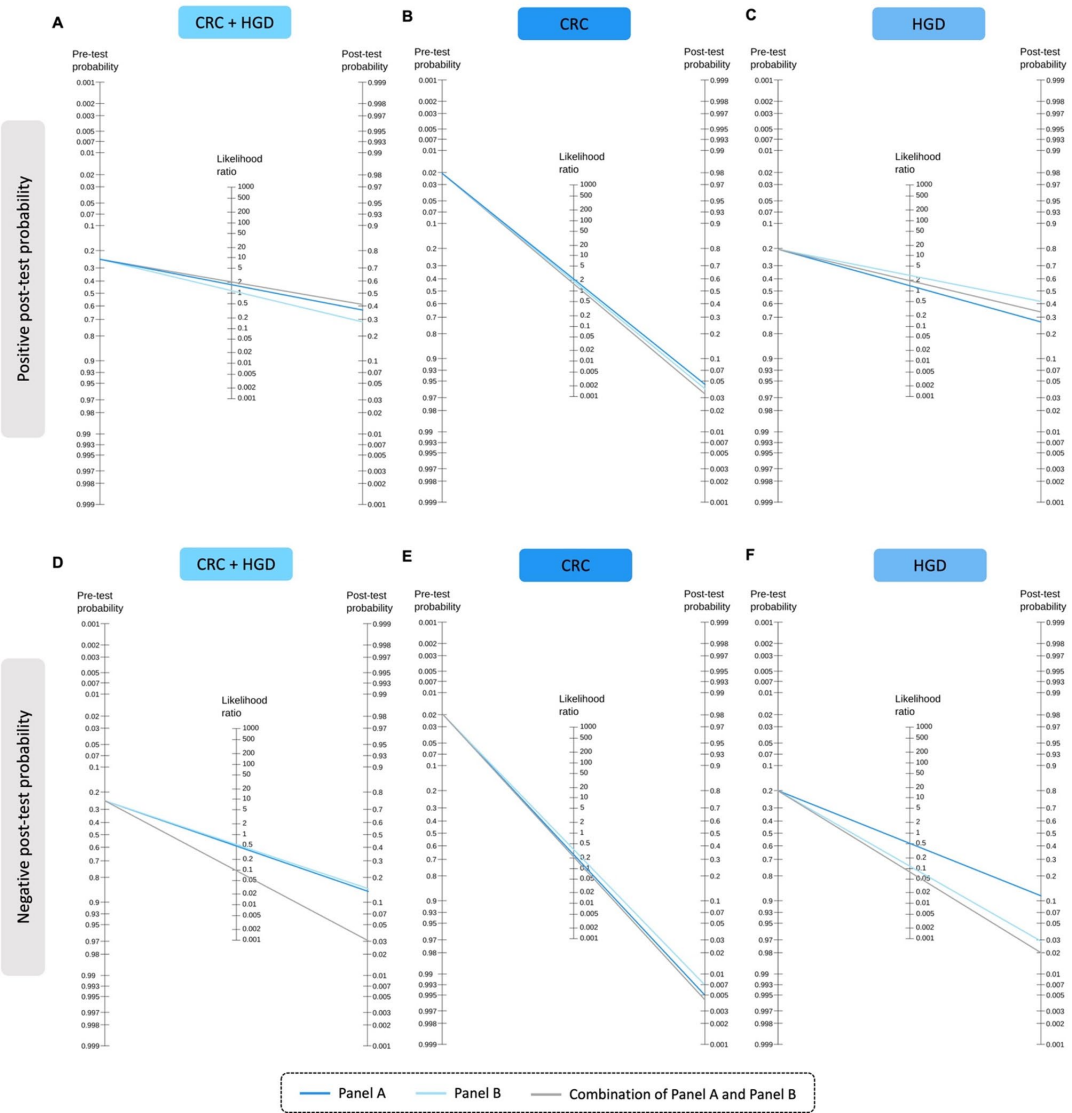
Fagan Nomogram showing the pre-test probability, the positive likelihood ratio, and the positive post-test probability for (**A**) CRC + HGD, (**B**) CRC, and (**C**) HGD lesions; and the pre-test probability, the negative likelihood ratio, and the negative post-test probability for (**D**) CRC + HGD, (**E**) CRC, and (**F**) HGD lesions for each panel, after a positive FOBT result. Panel A: miR-21-5p, miR-199a-5p, and age; Panel B: miR-21-5p, miR-199a-5p, miR-451a, age and gender. *HGD* High-Grade Dysplasia, *CRC* Colorectal Cancer.

The models had low positive likelihood ratios and post-test probabilities (Fig. 2 and Supplementary Table S2), indicating that a positive result in either stool-miRs panels—after a positive FOBT test—detects only around 35% of individuals with HGD and 4% of those with CRC.

On the other hand, negative likelihood ratios suggest that the miRs predictive models are effective in ruling out CRC and HGD lesions (negative likelihood ratios ≤ 0.5). Moreover, combining the outcomes of both panels resulted in a low post-test probability (0.5–3%) for all clinical conditions, reinforcing the potential of this approach to identify individuals with no lesions after a FOBT positive result.

## Discussion

Early detection and removal of colorectal lesions could prevent over 90% of CRC-related deaths^4^. However, current screening methods for CRC have several limitations. FOBT has suboptimal sensitivity for detecting premalignant and cancerous lesions, resulting in a significant number of false-negative results^5^. On the other hand, colonoscopy is an invasive procedure, with high costs and poor patient compliance^7,16^. Moreover, although FOBT specificity exceeds 90%, the low prevalence of CRC results in a low positive predictive value (PPV), which, in mass screening contexts, still yields a high absolute number of false positives, increasing the burden for healthcare systems^6^. Therefore, there is a pressing need to develop new non-invasive screening tools to overcome these limitations.

In recent years, there has been a research focus on miRs as promising powerful biomarkers for early CRC detection, mainly in blood samples^17,18^. However, recent findings suggest that miRs can be reliably detected in stool samples, demonstrating comparable reproducibility and stability to other biofluids, highlighting their potential as a non-invasive tool for CRC detection, namely by increasing the detection of CRC and HGD lesions and decreasing the false-positive rate^15,19,20^. In this study, we analysed the differential stool expression of eight candidate miRs, based on their efficacy in the diagnosis of CRC. Consistent with the literature, we found an upregulation for all miRs analysed in the HGD and CRC groups, with the exception of miR-451a, which was downregulated in HGD^10,15,21,22^. However, miR-199a-5p and miR-4516, firstly described in stool samples in this study, showed an expression pattern contrasting with reported findings in the literature for blood specimens^23,24^.

Regarding miRs’ clinical value, our results demonstrated miR-451a’s good performance to identify HGD lesions, indicating its potential to improve CRC screening. To our knowledge, this is the first time that miR-451a is assessed in HGD. However, Phua et al. previously reported a sensitivity of 87% for CRC discrimination, further indicating its promising clinical application^25^. Moreover, miR-199a-5p showed the best performance for CRC and HGD detection, confirming previous results in the literature and highlighting the potential clinical applicability of this miR^24^.

The literature supports the evidence that miR panels generally outperform individual miRs in detecting CRC and precancerous lesions, which was also observed in our study. Importantly, detecting HGD lesions is key for decreasing CRC mortality and clinical burden, however their low detection rate remains one of the main limitations of FOBT-based screening^26^. Notably, the combination of miR-21-5p, miR-199a-5p, and miR-451a with age and gender, two important risk factors for CRC, exhibited a sensitivity of 91% for HGD detection, making it one of the best described to date. In comparison, Pardini et al.^27^ reported a five-miR panel (miR-1246, miR-607-5p, miR-6777-5p, miR-4488, and miR-149-3p) combined with age and gender that showed a sensitivity of 61% for HGD detection. Similarly, Duran-Sanchon et al.^10^ described a combination of these two risk factors with the expression levels of miR-421 and miR-27a-3p, also with a sensitivity of 61% for HGD identification^10^. It is important to note that we were unable to determine the accuracy of our panels as standalone screening tools as we did not include FOBT-negative individuals in our study. However, our preliminary sensitivity results suggest that it may offer improved detection of individuals with HGD lesions compared to previously described miR signatures. Further studies with the inclusion of FOBT-negative individuals is essential to further validate these panels.

Regarding CRC identification, a panel with miR-21-5p, miR-199a-5p, and age improved the performance of individual miRs and demonstrated a good sensitivity value (88%) comparable to that described in the literature—Liu et al. combined miR-21-5p and miR-146-5p and achieved a sensitivity of 87%^28^. While blood-based panels including miR-21-5p outperformed stool samples (sensitivity > 95%)^29^, there is limited literature evaluating miRs’ performance (individually or in combination) for the detection of CRC and HGD in stool samples. In blood samples, Zhao et al. found that a miR-199a-5p and miR-627-5p panel identified these lesions with a sensitivity of 83%^24^. Herreros-Villanueva et al. reported a sensitivity of 85% for the identification of CRC and HGD lesions for a panel of six blood-based miRs^30^. In stool samples, Duran-Sanchon et al. reported a sensitivity of 67% for the detection of CRC and HGD on their predictive model (miR-421, miR-27a-3p, age, and gender)^10^. Our preliminary findings showed that by combining our predictive models we could enhance the detection of CRC and HGD lesions by detecting 92% of affected individuals.

Additionally, one of the main aims of our study was to evaluate whether a miR-based stool predictive model would improve screening as a secondary screening test following a positive FOBT result. The analysis of post-test probabilities (Supplementary Table S2) showed that a negative result in Panels A and B after a positive FOBT result corresponds to an approximately 3% probability of CRC or HGD lesions, i.e., demonstrating a high negative predictive value (NPV: ~97%), which highlights the potential of these panels to be used as a secondary screening test following a FOBT-positive result. Moreover, it is essential for a test to be applied following a FOBT-positive to have a high sensitivity (90–95%)^31^, namely for the identification of samples with CRC and HGD lesions. This is particularly important given that colonoscopy also has a therapeutic role due to its capacity for the removal of lesions^4^. However, while FOBT-based screening has a high specificity (> 90%) the absolute number of false positives can still be considerably high when applied to a large population, resulting in a significant burden for the healthcare system with the increased number of unnecessary colonoscopies performed^16^. Preliminary data from the screening program in the North of Portugal show that approximately 5% of the screened individuals have a FOBT-positive result, and of these individuals around 36% do not have lesions, 40% have LGD lesions, 22% have HGD lesions, and around 2% are diagnosed with cancer^6^—which means that at least 36% of the colonoscopies would be unnecessarily performed in patients without any lesion. When applying our miR-based test after a positive FOBT result— assuming a specificity of 52% for CRC + HGD detection versus NL as reference (data not shown),—this would correctly classify 52% of the FOBT false positives—thus reducing by 52% the number of unnecessary colonoscopies and reducing the burden on the healthcare system (Supplementary Fig. S3). Moreover, taking in consideration that LGD lesions have a slower and more gradual progression and often remain undetected for several years before progressing to HGD—which would then be detected if individuals remain under surveillance—, we can expect a further reduction in the number of colonoscopies performed. In this context—and considering a specificity of 40% (CRC + HGD versus NL + LGD scenario)—we would expect to reduce by 40% the number of unnecessary procedures in a universe that represents 76% of the whole FOBT-positive results (36% NL and 40% LGD). In addition, if we consider that the adherence to CRC screening programs is expected to increase in the next few years (from around 30% now to 80–90%), the number of procedures to perform could significantly increase^6^. Our results show that a CRC screening based on the FOBT followed-up by our miR-based tool could refine the triage process, thus reducing the clinical burden and ensuring that colonoscopies are focused on those individuals that truly require it.

Nevertheless, one of the limitations of our study was that we recruited patients in a hospital setting, with only FOBT-positive individuals performing a colonoscopy included in the study. Although this allowed us to include individuals with different type of lesions, it also limited the generalization of the results. The non-inclusion of FOBT-negative individuals thus restricted the clinical applicability of our miR panels to a FOBT clarification test before colonoscopy instead of a new standalone CRC screening tool. Furthermore, this preliminary work follows a case-control study design, which—although it allows us to assess the altered expression of miRs across the different types of lesions—means that the clinical groups sample sizes are not representative of real-world CRC screening programs as they are similar across groups. In this context, ongoing studies are focusing on further validating the potential of these miR panels in larger screening settings and including FOBT-negative results. This would address the limitations of this preliminary study and allow to assess the potential of these panels as a primary screening test.

In conclusion, this preliminary study highlights the potential use of stool miRs as promising biomarkers for the identification of premalignant and cancerous lesions and for the improvement of CRC screening. Here, the combination of miR-199a-5p and miR-451a with age and gender emerged as one of the most effective panels for the detection of HGD reported to date. Moreover, when combining both identified panels (i.e., positive in either or both), we achieved a sensitivity of 92% for the detection of CRC and HGD lesions. Applied as a secondary screening test after a positive FOBT, this approach could reduce unnecessary colonoscopies by ~ 40–52%, thereby reducing clinical burden and enhancing healthcare systems efficiency.

## Methods

### MicroRNA selection

**Fig. 3:**
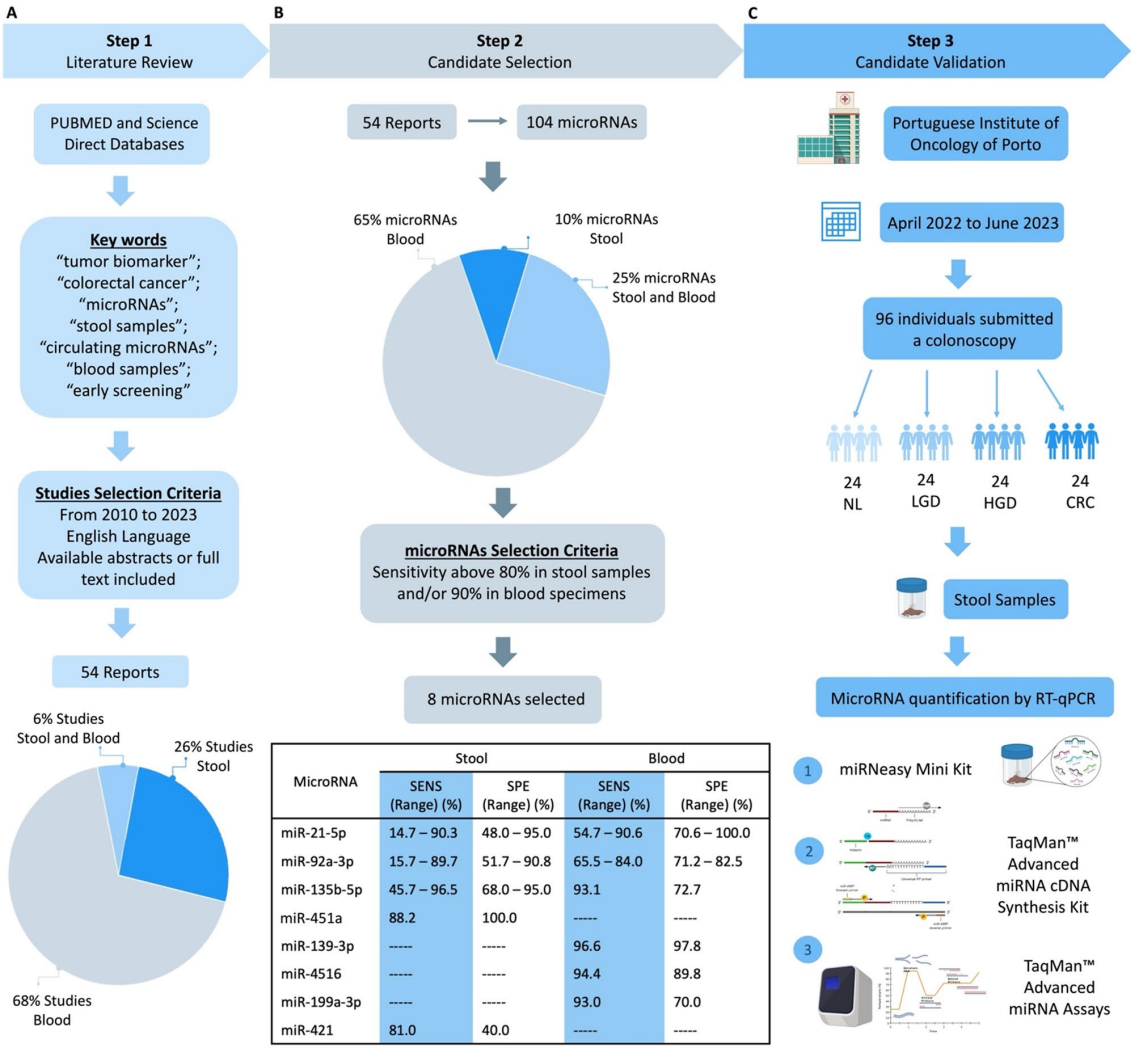
Stepwise strategy for selecting and assessing miRs potentially associated with CRC screening. (**A**) A comprehensive literature review was conducted to evaluate the reported miRs and their role in CRC detection using PUBMED and Science Direct databases. The 54 studies selected were published between 2010 and 2023, in English, and with abstracts or full text available. (**B**) Out of the 54 studies, 104 miRs associated with CRC screening were identified in different biological samples. After analysing the miRs expression profiles, eight miRs were selected, considering their sensitivity in stool and blood samples (the selected miRs and their sensitivity and specificity values are presented in the table). (**C**) A case-control study was conducted at IPO-Porto between April 2022 and June 2023. Stool Samples were collected from 96 individuals with colonoscopy results, divided into four groups based on the outcome (no lesion, low-grade dysplasia, high-grade dysplasia, and cancer). MiR quantification was performed using RT-qPCR with TaqMan™ Advanced miRNAs Assays. *NL* No Lesion, *LGD* Low-Grade Dysplasia, *HGD* High-Grade Dysplasia, *CRC* Colorectal Cancer.

Herein, taking into consideration our recently published comprehensive review^15^ on the role of miRs in early CRC screening, we selected miRs for CRC detection that demonstrated a sensitivity higher than (i) 80% in stool samples and/or (ii) 90% in blood samples (Fig. 3). We identified eight miRs: miR-21-5p, miR-92a-3p, miR-139-3p, miR-199a-5p, miR-4516, miR-451a, miR-421, and miR-135b-5p.

### Patients recruitment and stool samples collection

A case-control study was implemented at the Gastroenterology Department of the Portuguese Oncology Institute of Porto (IPO-Porto), recruiting individuals with a positive FOBT result who underwent colonoscopy. Between April 2022 and July 2023, stool specimens from the recruited individuals were freshly collected in a sealed plastic container on the day prior to the colonoscopy. The stool specimens where then coded, homogenised, and stored at -80 °C, as previously described^32^. A total of 96 stool samples were included, divided equally into four groups according to the colonoscopy outcome: individuals (i) with no lesions (NL), (ii) with at least one low-grade dysplasia lesion (LGD), (iii) with at least one high-grade dysplasia lesion (HGD), and (iv) diagnosed with CRC. In detail, and according to WHO guidelines: LGD lesions show preserved architecture and nuclear polarity, with mild atypia and basal mitoses; while HGD lesions show loss of polarity, marked atypia, mitoses at all levels, and complex glandular structure, indicating a greater potential for malignancy^33^. Each individual was assigned to the group corresponding to the highest-grade lesion present at least once at the time of diagnosis. Subsequently, we also grouped these individuals into two groups: (i) those with non-relevant findings (NL + LGD, since lesion progression is slow and gradual and may remain undetectable for several years until it progresses to HGD lesions or CRC); and (ii) individuals with HGD lesions or CRC. As in a case-control study the number of samples in each group should be identical, sample selection followed a sequential approach, with the first 24 participants from each group included in the study. The study was approved by IPO-Porto’s Ethical and Data Protection Committee (reference: CES 22/022, 10 February 2022) and followed international guidelines. All participants signed an informed consent form.

### MicroRNA isolation and cDNA synthesis

Total RNA was extracted from stool samples using the miRNeasy Mini Kit (Qiagen, Germany) according to the manufacturer’s protocol. RNA concentration and purity were assessed by spectrophotometry on the NanoDrop Lite (Thermo Scientific^®^, Waltham, MA, USA). Then, 160 ng of RNA was converted into cDNA using the TaqMan™ Advanced miRNA cDNA Synthesis Kit (Applied Biosystems^®^, Foster City, CA, USA), in accordance with the manufacturer’s instructions.

### MicroRNA quantification by RT-qPCR

MiR expression levels were determined by quantitative real-time PCR analysis, using a 7500 Real-Time PCR System (Applied Biosystems^®^, Foster City, CA, USA). The PCR reaction consisted of a volume of 10 µL, containing TaqMan™ Advanced miRNA Assays probes (hsa-miR-21-5p: 477975_mir; hsa-miR-92a-3p: 477827_mir; hsa-miR-139-3p: 477906_mir; hsa-miR-199a-5p: 478231_mir; hsa-miR-4516: 478303_mir; hsa-miR-451a: 478107_mir; hsa-miR-421: 478088_mir; hsa-miR-135b-5p: 478582_mir), TaqMan™ Fast Advanced Master Mix (Applied Biosystems^®^, Foster City, CA, USA), and 2.5 µL of cDNA. For normalisation of miR expression, hsa-miR-345-5p (478366_mir) was used as endogenous control. This reference gene was selected based on previous literature reports due to its stability among CRC samples^34,35^. Prior to the full analysis, the stability of this gene as an endogenous control in stool samples was confirmed in 20 samples and assessed using the NormFinder Software (version 0.953, Excel add-in, Department of Molecular Medicine, Aarhus, University Hospital, Denmark). The amplification conditions included an initial holding stage at 95 °C for 20 s, followed by 50 cycles at 95 °C for 3 s and 60 °C for 30 s. Three technical replicates were performed for each sample, and the mean value was used to calculate the miR expression levels using the 2^−△Ct^ formula.

### Data processing and statistical analysis

Data analysis was performed using SPSS (version 28.0, IBM, Inc., Chicago, USA) and GraphPad Prism 10.0 (GraphPad Software, Inc., San Diego, California, USA). A Mann-Whitney test was performed for continuous variables, as data did not follow a normal distribution, and a *P*-value under 0.05 was considered statistically significant.

The clinical value of each miR for detecting the presence of HGD and/or cancer lesions was assessed using receiver operating characteristic (ROC) curves. The optimal threshold cutoff value was determined from the ROC curve by identifying the point where the Youden’s Index (= sensitivity + specificity − 1) was maximised. This cutoff value was used to categorise each miR expression level as either ‘Low’ or ‘High’. Subsequently, we assigned a value of 0 or 1 for the ‘Low’ and ‘High’ levels, respectively (with the exception of miR-451a, since low levels were associated with HGD lesions). The Area Under the Curve^36^ value was classified as: AUC lower than 0.5 – no discrimination; AUC 0.6–0.7 – acceptable discrimination; AUC 0.7–0.8 – moderate discrimination; AUC 0.8–0.9 – excellent discrimination; and AUC 0.9–1.0 – outstanding discrimination^37^. It should be noted that miR-139-3p (undetected in stool samples) and miR-421 (expressed in only 20% of cases) were excluded from these analyses. To assess the clinical value of the evaluated miRs in combination, a stepwise multivariate logistic regression with backward elimination (with a *P*-value for retention of 0.1) was conducted. Clinical variables such as age and gender were also included in the analysis. The variables retained were used to produce a score for each model as the sum of the expression level of each variable multiplied by the corresponding β coefficient. The clinical value of the calculated scores was assessed as previously described for each miR individually. Additionally, to analyse the clinical applicability of each model, the likelihood ratio and the post-test probability were calculated using ROC curves and Fagan nomograms were performed.

## Supplementary Information

Below is the link to the electronic supplementary material.

Supplementary Material 1

Supplementary Material 2

Supplementary Material 3

Supplementary Material 4

## Data Availability

The data generated in this study are included in the main article or supplementary material.
