# Supplementary figures and images for: A preliminary assessment of a stool-based microRNA profile for early colorectal cancer screening
Source: Sci Rep. 2025 Aug 5;15:28597. doi: 10.1038/s41598-025-14485-z (PMC12325799; doi:10.1038/s41598-025-14485-z)

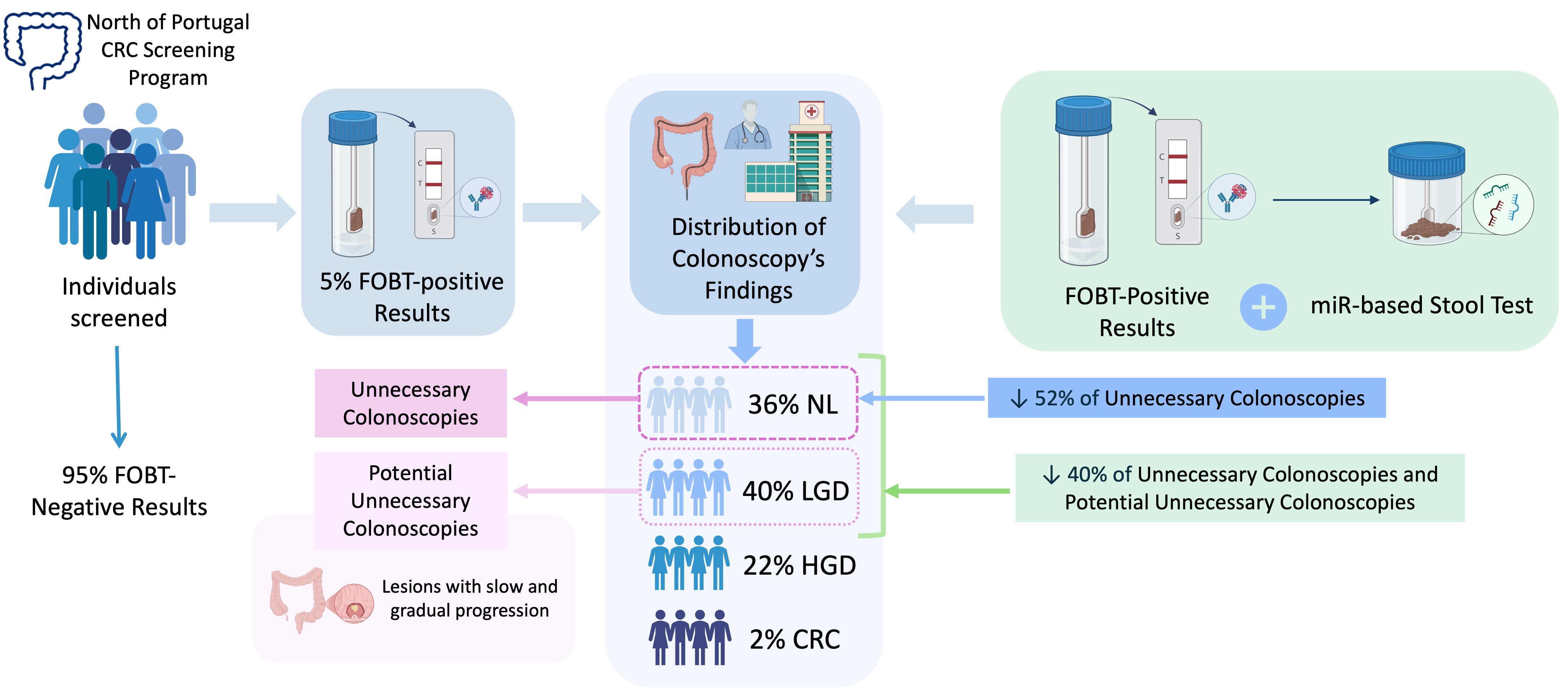

Supplement: Supplementary file 1 — Supplementary Material 1 [file 41598_2025_14485_MOESM1_ESM.jpg]
