# Supplementary material for: A preliminary assessment of a stool-based microRNA profile for early colorectal cancer screening
Source: Sci Rep. 2025 Aug 5;15:28597. doi: 10.1038/s41598-025-14485-z (PMC12325799; doi:10.1038/s41598-025-14485-z)
Supplement: Supplementary file 4 — Supplementary Material 4 [file 41598_2025_14485_MOESM4_ESM.docx]

**Table S1:** Performance Characteristics of Stool miRs to identify Individuals from Different Clinical Groups

| miR | Clinical  Groups | AUC  (95% CI) | Youden Index | |
| --- | --- | --- | --- | --- |
|  |  |  | SENS (%)  (95% CI) | SPEC (%)  (95% CI) |
| miR-21-5p | CRC + HGD*^a^* | 0.623  (0.507 – 0.739) | 41  (28 – 56) | 86  (73 – 93) |
|  | CRC*^b^* | 0.656  (0.495 – 0.817) | 61  (41 – 78) | 73  (52 – 87) |
|  | HGD*^b^* | 0.634  (0.469 – 0.800) | 65  (45 – 81) | 64  (43 – 80) |
|  | LGD*^b^* | 0.548  (0.369 – 0.727) | 57  (37 – 76) | 73  (52 – 87) |
| miR-92a-3p | CRC + HGD*^a^* | 0.620  (0.507 – 0.733) | 56  (42 – 69) | 68  (54 – 80) |
|  | CRC*^b^* | 0.592  (0.427 – 0.758) | 58  (39 – 76) | 65  (45 – 81) |
|  | HGD*^b^* | 0.609  (0.444 – 0.773) | 75  (55 – 88) | 57  (37 – 74) |
|  | LGD*^b^* | 0.538  (0.371 – 0.705) | 46  (28 – 65) | 70  (49 – 84) |
| miR-199a-5p | CRC + HGD*^a^* | 0.648  (0.536 – 0.759) | 71  (57 – 82) | 60  (45 – 72) |
|  | CRC*^b^* | 0.627  (0.465 – 0.789) | 71  (51 – 85) | 57  (37 – 74) |
|  | HGD*^b^* | 0.656  (0.496 – 0.816) | 92  (74 – 98) | 39  (22 – 59) |
|  | LGD*^b^* | 0.502  (0.331 – 0.673) | 83  (64 – 93) | 39  (22 – 59) |
| miR-4516 | CRC + HGD*^a^* | 0.649  (0.538 – 0.761) | 55  (41 – 69) | 74  (60 – 85) |
|  | CRC*^b^* | 0.576  (0.410 – 0.743) | 79  (60 – 91) | 39  (22 – 59) |
|  | HGD*^b^* | 0.592  (0.423 – 0.761) | 65  (45 – 81) | 70  (49 – 84) |
|  | LGD*^b^* | 0.598  (0.433 – 0.763) | 96  (80 – 100) | 30  (16 – 51) |
| miR-451a | CRC + HGD*^a^* | 0.522  (0.386 – 0.658) | 55  (40 – 69) | 55  (38 – 71) |
|  | CRC*^b^* | 0.583  (0.399 – 0.767) | 43  (24 – 63) | 76  (53 – 90) |
|  | HGD*^b^* | 0.706  (0.533 – 0.879) | 63  (41 – 81) | 76  (53 – 90) |
|  | LGD*^b^* | 0.668  (0.468 – 0.868) | 79  (52 – 92) | 53  (31 – 74) |
| miR-135b-5p | CRC + HGD*^a^* | 0.581  (0.462 – 0.699) | 84  (71 – 92) | 37  (25 – 51) |
|  | CRC*^b^* | 0.542  (0.381 – 0.712) | 100  (85 – 100) | 17  (7 – 37) |
|  | HGD*^b^* | 0.558  (0.380 – 0.726) | 78  (58 – 90) | 39  (22 – 59) |
|  | LGD*^b^* | 0.552  (0.381 – 0.709) | 57  (37 – 74) | 65  (45 – 81) |

Abbreviations: miR- microRNA; LGD – Low-Grade Dysplasia; HGD – High-Grade Dysplasia; CRC – Colorectal Cancer; HGD + CRC – Advanced Lesions; NR – Non-relevant Lesions; AUC: Area under Curve; SENS: Sensitivity; SPEC: Specificity; CI – Confidence Intervals.  ^a^ Negative Category: individuals with non-relevant findings in colonoscopy; ^b^ Negative Category: individuals without lesions detected in colonoscopy.

**Table S2:** Diagnostic characteristics of each stool-based panel and the score combination of two miRs panel

| Endpoint | Probability Pre-test | miRs Combination | Positive Likelihood Ratio | Positive Post-test Probability (%) | Negative Likelihood Ratio | Negative Post-test Probability (%) |
| --- | --- | --- | --- | --- | --- | --- |
| CRC + HGD | 24% | Panel A | 2.2 | 41 | 0.5 | 12.8 |
|  |  | Panel B | 1.3 | 29 | 0.5 | 14.22 |
|  |  | Panel A + B | 1.9 | 38 | 0.1 | 3.1 |
| CRC | 2% | Panel A | 1.7 | 3.3 | 0.3 | 0.51 |
|  |  | Panel B | 1.9 | 3.7 | 0.4 | 0.73 |
|  |  | Panel A + B | 1.8 | 3.6 | 0.2 | 0.47 |
| HGD | 22% | Panel A | 1.4 | 29 | 0.5 | 12.8 |
|  |  | Panel B | 2.6 | 43 | 0.1 | 3.4 |
|  |  | Panel A + B | 2.0 | 36 | 0.1 | 2.1 |

Panel A: miR-21-5p, miR-451a, miR-199a-5p and age; Panel B: miR-21-5p, miR-451a, miR-199a-5p, age and gender.

**
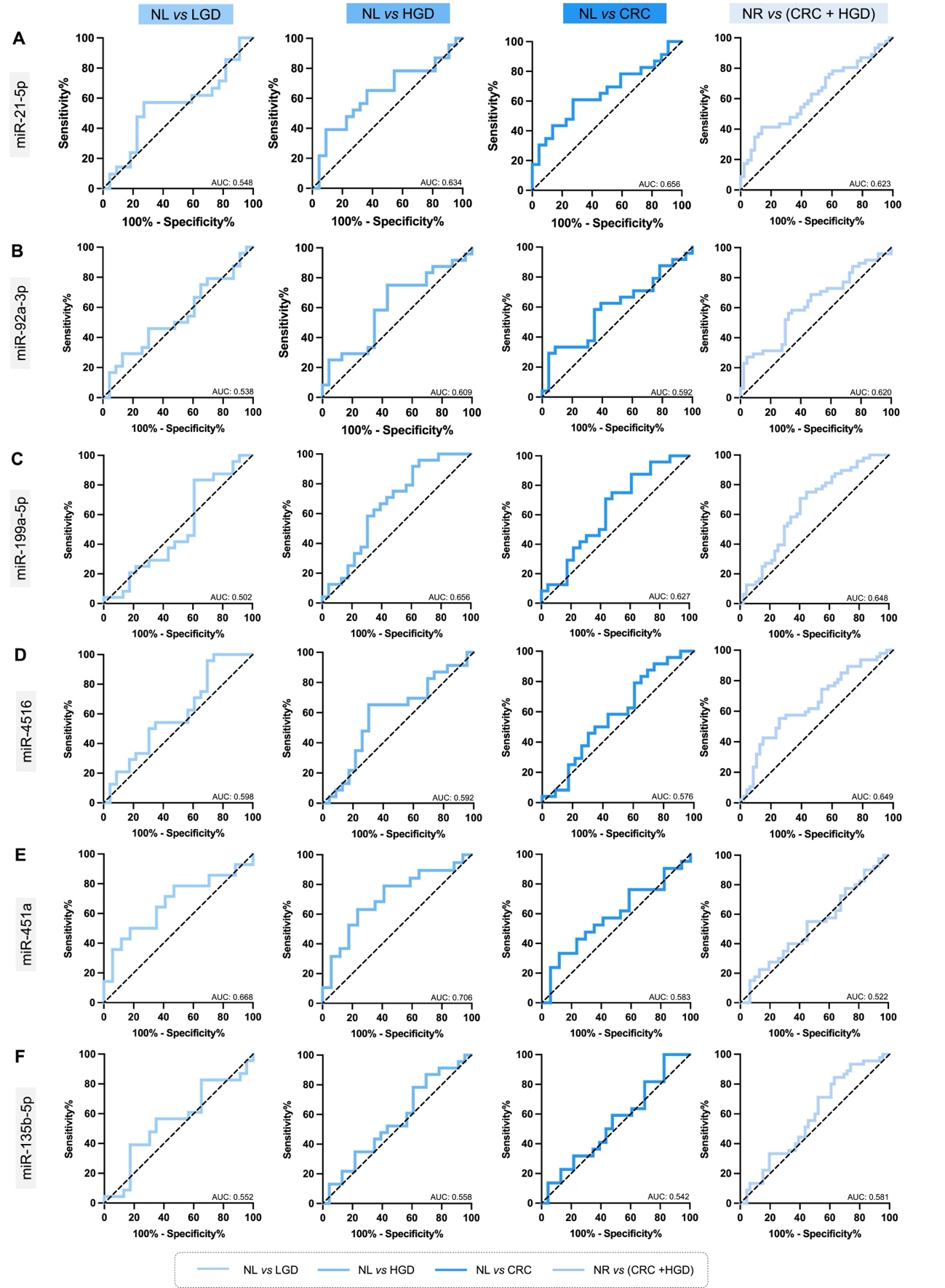
**

**Figure S1** ROC analysis and AUC values of stool miR-21-5p **(A)**, miR-92a-3p **(B)**, miR-199a-5p **(C)**, miR-4516 **(D)**, miR-451a **(E)** and miR-135b-5p **(F)**. Abbreviations: NL – No Lesion; LGD – Low-Grade Dysplasia, HGD – High Grade Dysplasia; CRC – Colorectal Cancer; NR – Non-relevant Findings in Colonoscopy (NL + LGD).

**
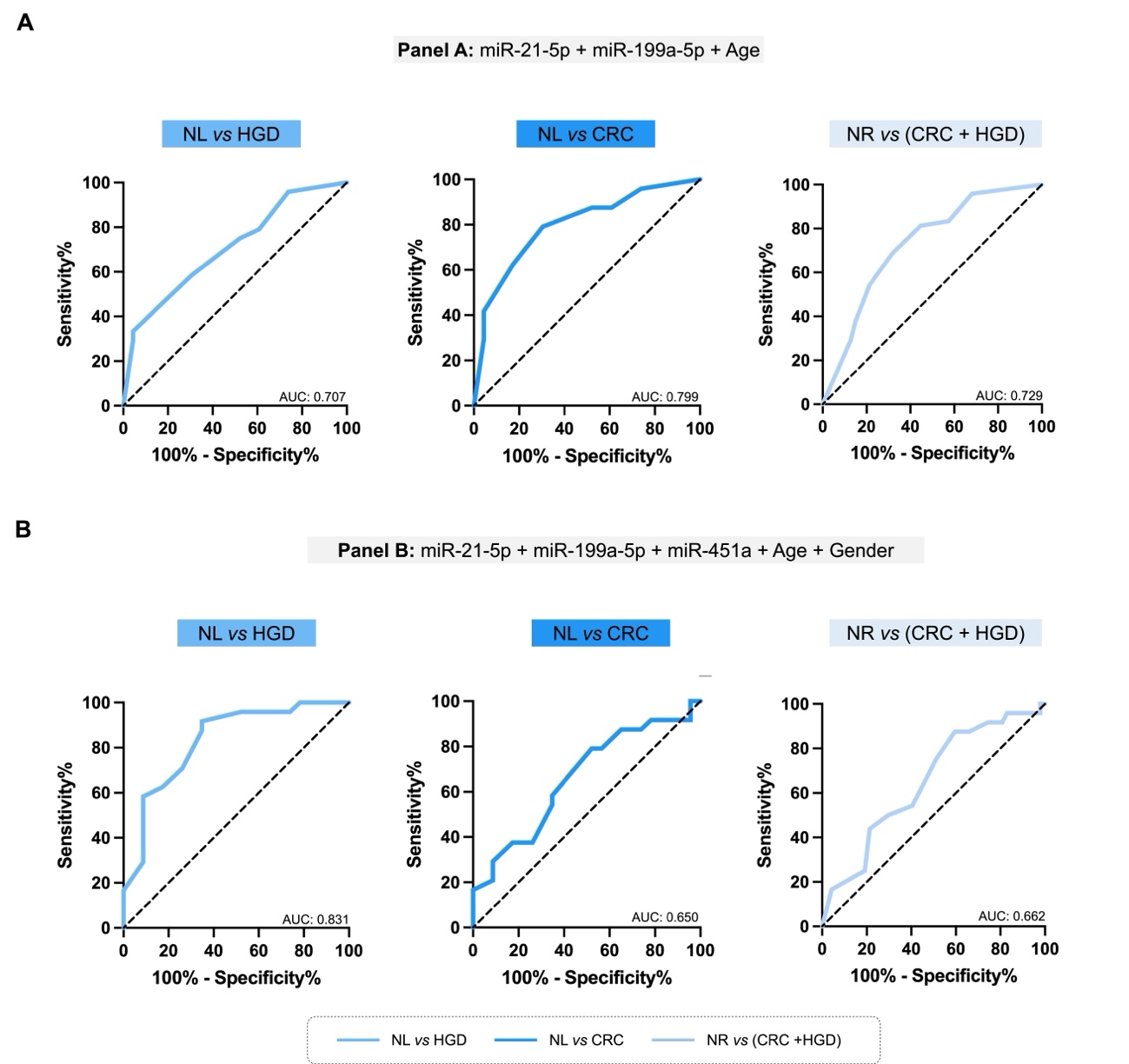
**

**Figure S2** ROC analysis of stool-based microRNAs panels. AUC values of miR-21-5p, miR-199a-5p and Age in combination for HGD, CRC and AL **(A)**, and miR-199a-5p, miR-451a, Age and Gender for HGD, CRC and AL **(B)** Abbreviations: NL – No Lesion; LGD – Low-Grade Dysplasia, HGD – High Grade Dysplasia; CRC – Colorectal Cancer; NR – Non-relevant Findings in Colonoscopy (NL + LGD).


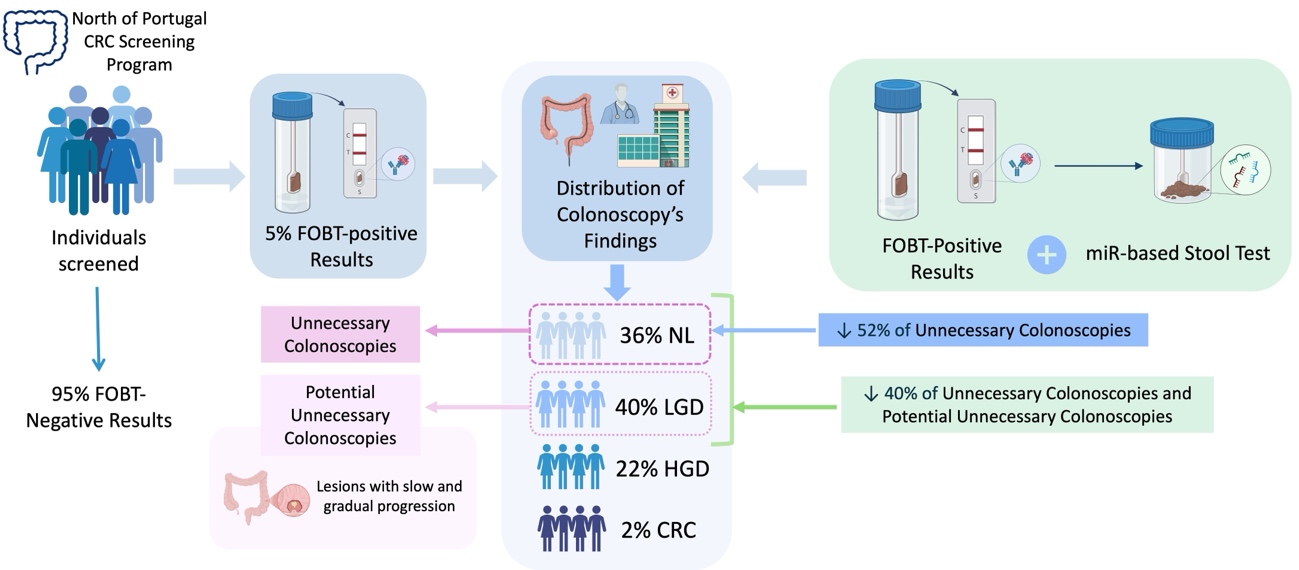


**Figure S3** Schematical representation of the number of unnecessary colonoscopies and potentially unnecessary colonoscopies following a positive FOBT-based screening test, compared with the application of a miR-based stool test after positive FOBT-positive Teste. Abbreviation: FOBT – Fecal Occult Blood Test; miR – microRNAs; NL – No Lesion; LGD – Low-grade dysplasia; HGD – High-grade dysplasia; CRC – Colorectal Cancer.
